# Myelin and lymphocyte protein serves as a prognostic biomarker and is closely associated with the tumor microenvironment in the nephroblastoma

**DOI:** 10.1002/cam4.4542

**Published:** 2022-01-13

**Authors:** Cheng Su, Rongzhi Huang, Zhenyuan Yu, Jie Zheng, Fengling Liu, Haiqi Liang, Zengnan Mo

**Affiliations:** ^1^ Department of Pediatric Surgery The First Affiliated Hospital of Guangxi Medical University Nanning China; ^2^ Department of Urology The First Affiliated Hospital of Guangxi Medical University Nanning China; ^3^ Guangxi Medical University Nanning China; ^4^ Guangxi Key Laboratory for Genomic and Personalized Medicine Guangxi Key Laboratory of Colleges and Universities Nanning China; ^5^ Institute of Urology and Nephrology The First Affiliated Hospital of Guangxi Medical University Nanning China; ^6^ Guangxi Collaborative Innovation Center for Genomic and Personalized Medicine Guangxi Medical University Nanning China

**Keywords:** GSEA, *MAL*, methylation, tumor microenvironment, Wilms' tumor

## Abstract

Nephroblastoma, also known as Wilms' tumor (WT), is the most common renal tumor that occurs in children. Although the efficacy of treatment has been significantly improved by a series of comprehensive treatments, some patients still have poor prognosis. Myelin and lymphocyte (*MAL*) protein, a highly hydrophobic integrated membrane‐bound protein, has been implicated in many tumors and is also closely linked to kidney development. However, the relationship between *MAL* and WT has not yet been elucidated. Therefore, we attempted to evaluate the feasibility of *MAL* as a promising prognosis factor for WT. The differential expression of *MAL* was investigated using TARGET database and was verified using the Gene Expression Omnibus database and real‐time quantitative PCR. The prognostic ability of *MAL* was determined using Kaplan–Meier and Cox regression analyses. Pearson correlation analysis was applied to explore the relationship between *MAL* expression and methylation sites. The ESTIMATE and CIBERSORT algorithms showed that *MAL* expression was associated with the WT tumor microenvironment. Gene Set Enrichment Analysis (GSEA) indicated that multiple signaling pathways closely associated with tumorigenesis were differentially enriched between the high‐ and low‐*MAL* groups. In conclusion, our study comprehensively explored the potential *of MAL* as a prognosis factor for WT. Meanwhile, we also demonstrated that *MAL*, as a prognostic factor for WT, may be closely related to the tumor microenvironment.

## Graphical abstract

MAL serves as a prognostic factor and closely associates with tumor microenvironment in WT. Our study comprehensively explored the potential of MAL as a prognosis factor for WT. Meanwhile, we also demonstrated that MAL, as a prognostic factor for WT, may be closely related to the tumor microenvironment.

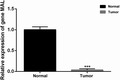

## 1 INTRODUCTION

Nephroblastoma, also known as Wilms' tumor (WT), is the renal tumor with the highest incidence in children, accounting for 90% of all childhood renal tumors.^1^ It has been shown that about 1 in 10,000 children have had this disease, with the highest incidence at the age of 3.^2^ At present, the overall survival (OS) rate of patients with WT has increased to more than 90% through a series of comprehensive standard therapies.^3^, ^4^ However, some patients still have poor prognosis because of cancer recurrence and metastasis,^5^ and a quarter of survivors experience severe chronic diseases associated with the antitumor therapy they receive.^6^ Therefore, new and promising prognosis biomarkers are needed to explore for patients with WT and provide a reference for the implementation of clinical decision‐making.

As a primitive, pluripotent embryonic renal precursors malignancy, a large number of studies have shown that the occurrence of WT was closely related to early renal dysplasia.^7^ The kidneys began to develop through a series of complex tissue interactions between mesoderm derivatives around the fifth week of pregnancy. In this process, the transformation of mesenchymal progenitor cells into epithelial cells was crucial, which was finely regulated by a gene network composed of a series of genes. Mutations of genes in this process, such as the WT1 gene and related genes in the WNT pathway, often resulted kidney dysplasia thus caused a series of kidney related diseases.^8^, ^9^, ^10^, ^11^ Therefore, the normal development of the kidney was of great significance for the exemption of kidney‐related diseases. Frank et al.^12^ found that during the whole development of kidney, the high expression level of *MAL* would be maintained until adulthood. And *MAL* played a role of structural protein in the apical membrane of specialized epithelium such as renal tubules. Its functions of differentiation and transportation may play an important role in the development of kidney.^12^, ^13^ *MAL* had been reported to play an important role in a variety of tumors,^14^, ^15^, ^16^, ^17^ but the role of *MAL* gene in nephroblastoma had not been reported yet. Therefore, we perceived that it was necessary to explore the features of *MAL* in WT and conduct further studies.

In this study, we tried to clarify the relationship between *MAL* expression and the prognosis of WT through related bioinformatics methods, and at the same time explored its relationship with tumor microenvironment and related signaling pathways. The results from our research were expected to provide new perspectives for exploring the prognosis factors for WT and guide for the implementation of clinical decision‐making.

## 2 MATERIALS AND METHODS

### 2.1 Differential expression of *MAL* between WT and normal tissues

First, the fragments per kilobase of exon model per million mapped reads (FPKM) RNA‐seq data of 124 primary WT and six adjacent normal tissues were download by using TCGAbiolinks R package. Further, corresponding clinical data were acquired from UCSC Xena (https://xenabrowser.net). Next, the Emsembl database (http://asia.ensembl.org/index.html) was used to convert Ensembl IDs into gene symbols. The expression file of the *MAL* gene was extracted for further analysis. Differential expression of *MAL* between WT and adjacent normal tissues was identified using a *t*‐test. Finally, Gene Expression Omnibus database (GEO. http://www.ncbi.nlm.nih.gov/geo/) was used to verify the accuracy of the results, including GSE2712, GSE1115, and GSE73209. Detailed information of these validation datasets is shown in Table 1.

**TABLE 1:** Information of three GSE validation datasets

GSE datasets	Platform	Annotation package	Sample
GSE11151	GPL570	Hgu133plus2.db	ANK 3 FNK 2 WT 4
GSE2712	GPL96	Hgu133a.db	FNK 3 WT 18
GSE73209	GPL10556	IlluminaHumanv4.db	ANK 2 FNK 4 WT 32

Abbreviation: ANK, adult normal kidney; FNK, fetal normal kidney; WT, Wilms' tumor.

### 2.2 Prognostic value analysis

The expression profile was processed in log2 (normalized value + 1) data format. Survival analysis was performed using the survminer R package. The log‐rank test was used to confirm statistical significance, and the basis of grouping was dependent on the median value of *MAL* expression. The comparison between *MAL* expression and other clinical characteristics on the survival impact of patients with WT was explored by univariate and multivariate Cox analyses (age, gender, and stage).

### 2.3 Correlation analysis between *MAL* expression and methylation

From the Target database (https://ocg.cancer.gov/programs/target), the methylation data of WT were obtained. Methylation sites of the *MAL* gene were extracted for further analysis. The relationship between *MAL* expression and methylation was explored using the Pearson correlation test. The survival impact of methylation sites on patients with WT was explored by KM analysis. The basis of grouping was dependent on the median *β* value.

### 2.4 Exploration of relationship between *MAL* expression and tumor microenvironment

We further explored the relationship between *MAL* expression and tumor microenvironment in order to understand the difference of tumor microenvironment between high and low‐*MAL* groups. The tumor microenvironment was identified as the cellular environment where cancerous cells were present, which was composed of a series of cell types, including mesenchymal cells, immune cells, inflammatory mediators, endothelial cells, and extracellular matrix (ECM) molecules.^18^ The stromal score, immune score, ESTIMATE score, and tumor purity of each sample were calculated in the R platform using the ESTIMATE algorithm. The difference between high and low‐*MAL* groups was further compared using the Wilcoxon test. KM analysis was performed to explore the survival influence of tumor microenvironment‐related scores. The basis of grouping was determined by its median value.

The infiltration fraction of 22 immune cell types in each sample was estimated by performing the CIBERSORT algorithm. The Wilcoxon test was conducted to compare the difference between high and low‐*MAL* groups.

### 2.5 Differential signaling pathways demonstrate through GSEA between the high and low‐*MAL* groups

The differentially activated signaling pathways between the high‐ and low‐*MAL* groups were explored in the R platform. The ordered gene list was identified using the edgeR R package, and Gene Set Enrichment Analysis (GSEA) was performed using the gseKEGG function of clusterProfiler R package.^19^

### 2.6 Real‐time quantitative PCR analysis

We performed real‐time quantitative PCR (RT‐qPCR) in vitro to show the expression of *MAL* in WT. The clinical specimens were from three adjacent normal tissues and three WT tissues who had been pathologically identified at the Department of Pathology, First Affiliated Hospital of Guangxi Medical University, from January 2019 to December 2019, and processed suitably. RT‐qPCR was performed using PC33‐2 in an ABI 7500 cycler (Applied Biosystems, Aidlab Biotechnologies Co., Ltd). The differential expression of *MAL* in WT tissues relative to the adjacent normal tissues was calculated using the 2^−ΔΔ^ *Ct* method, and GAPDH was identified as the internal control. The primers of *MAL* and GAPDH were synthesized by Aidlab Biotechnologies and the sequences were as follows: *MAL*: Forward 5′‐CGACTTGCTCTTCATCTTTGAG‐3′ and Reverse 5′ ATGTACAG GATGATCAAGGTGG‐3′; GAPDH: Forward 5′‐ AGAAGGCTGGGGCTCATTTG‐3′ and reverse 5′‐ AGGGGCCATCCACAGTC TTC‐3′. All experiments were repeated at least three times.

### 2.7 Statistical analysis

All statistical analyses were carried out using the R platform (version: 3.61, http://www.r‐project.org/ ). The relationship between *MAL* expression and pathological features was analyzed using the Kruskal–Wallis and Wilcoxon rank tests. The minifi R package was used to process the original data and calculate the *β* value. The IlluminaHumanMethylation450kanno.ilmn12.hg19 R package was used for the conversion of gene symbols. Two‐tailed *p* value < 0.05 was considered statistically significant in all statistical analysis: *p < 0.05, **p < 0.01, ***p < 0.001, and ****p < 0.0001.

## 3 RESULTS

### 3.1 Differential expression in *MAL* between WT and normal tissues

**FIGURE 1:**
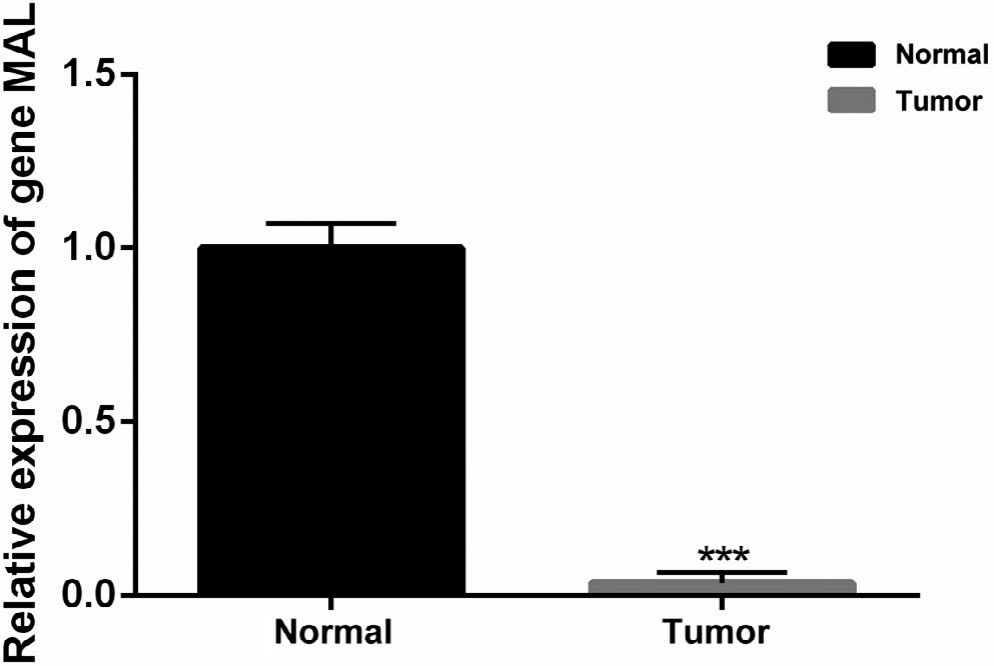
The RT‐qPCR verifies the expression of gene *MAL* in Wilms' tumor and adjacent normal tissues

The results of RT‐qPCR analysis are shown in Figure 1, and the expression of *MAL* was lower in WT than in adjacent normal tissues (*p* < 0.05, Figure 1), which showed the differential expression of *MAL* in adjacent normal tissues and WT in vitro.

**FIGURE 2:**
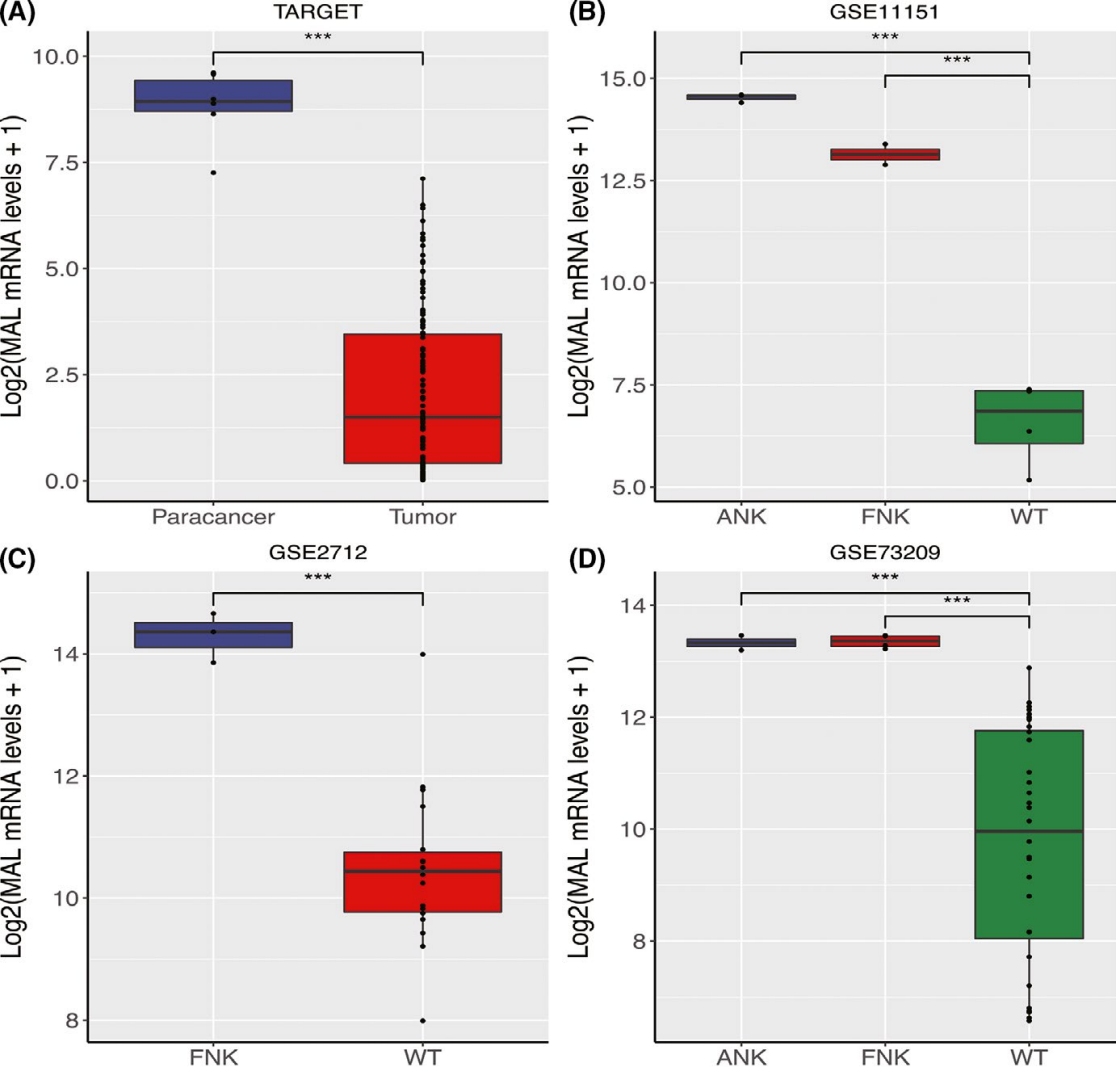
Boxplots of the analysis of *MAL* expression levels in WT and adjacent normal tissues. Four datasets indicate that the level of expression of *MAL* is lower in WT compared with adjacent normal tissues (*p* < 0.05). (A) TARGET datasets (B) GSE11151 (C) GSE2712 (D) GSE73209. *means *p* < 0.05, **means *p* < 518 0.01, and ***means *p* < 0.001. An ns means not statistically significant. ANK, adult normal kidney; FNK, fetal normal kidney; WT, Wilms' tumor

**FIGURE 3:**
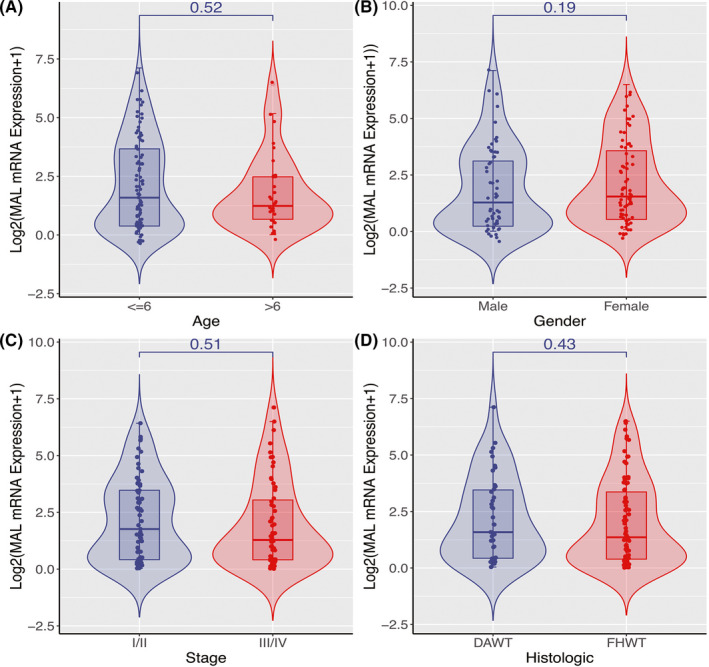
*MAL* expression and clinical characteristics (A) Age (B) Gender (C) Stage (D) Histologic

The expression of *MAL* in the TARGET, GSE2712, GSE11151, and GSE73209 datasets also showed significant statistical differences (Figure 2A–D, *p* < 0.05). *MAL* was inadequately expressed in WT, and it was not significantly correlated with age, gender, stage, and histologic type (Figure 3A–D).

### 3.2 Survival outcomes and univariate COX analysis

**FIGURE 4:**
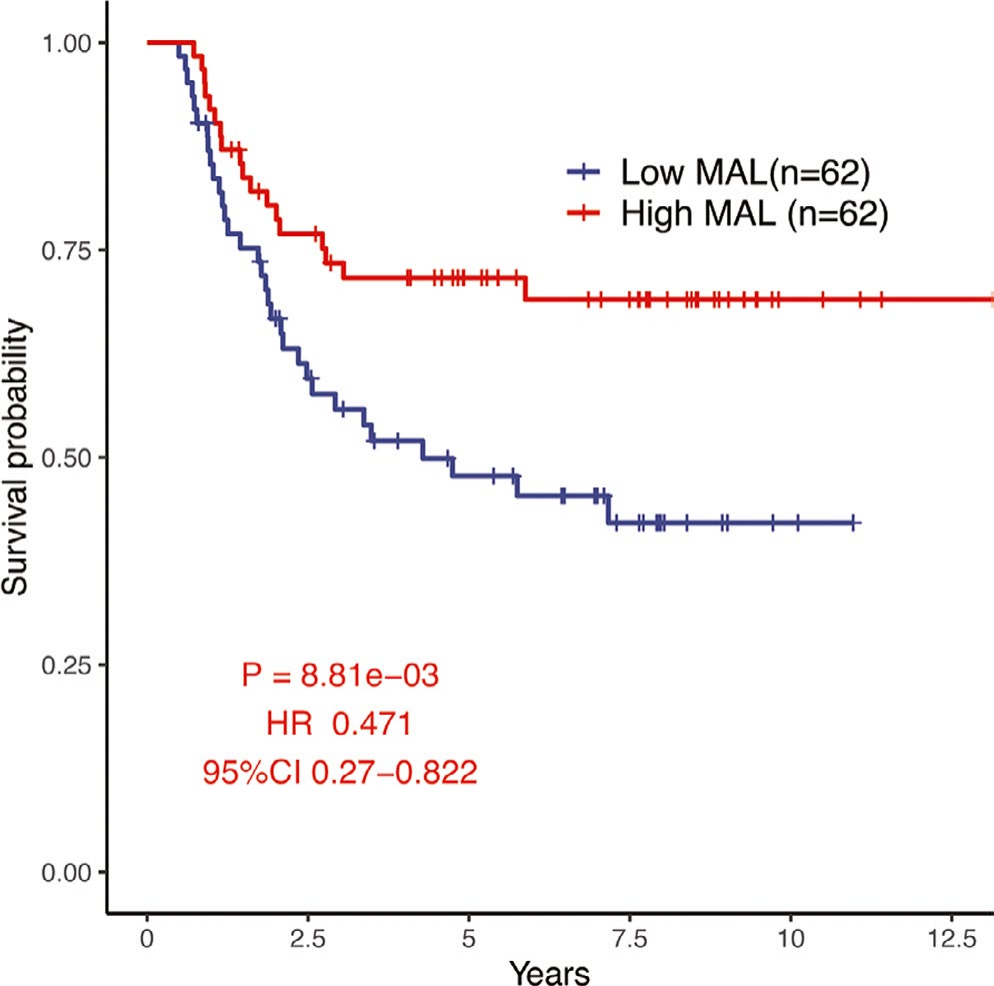
Influence of *MAL* expression on OS in patients with WT in RNA‐Seq cohort

KM analysis showed that the low‐*MAL* group had worse prognosis than the high‐*MAL* group in WT (*p* = 8.81e‐03, HR = 0.471, 95% CI: 0.27–0.822, Figure 4). In addition, univariate and multivariate Cox analyses proved that *MAL* was an independent prognostic factor for overall survival (OS), with an HR of 0.582 (*p* = 2.35E‐02, Table 2).

**TABLE 2:** The results of univariate and multivariable Cox regression analyses

Characteristics	Univariate Cox analysis		Multivariate Cox analysis	
	HR (95% CI)	*p* value	HR (95% CI)	*p* value
Age				
≤6	Reference			
>6	0.559 (0.26–1.19)	1.33E−01		
Gender				
Male	Reference		Reference	
Female	0.571 (0.33–1)	4.86E‐02	0.467 (0.26–0.82)	8.39E‐03
Stage				
I/II	Reference		Reference	
III/IV	3.151 (1.74–5.72)	1.60E‐04	3.462 (1.89–6.35)	6.06E‐05
Histologic				
DAWT	Reference			
FHWT	0.894 (0.5–1.61)	7.08E‐01		
*MAL*				
Low	Reference		Reference	
High	0.47 (0.26–0.84)	1.05E‐02	0.511 (0.29–0.91)	2.35E‐02

Abbreviations: CI, confidential interval; DAWT, Diffuse Anaplastic Wilms Tumors; FHWT, Favorable Histology Wilms Tumors; HR, hazard ratio; *MAL*, Myelin and lymphocyte.

### 3.3 Correlation analysis between *MAL* expression and methylation

**FIGURE 5:**
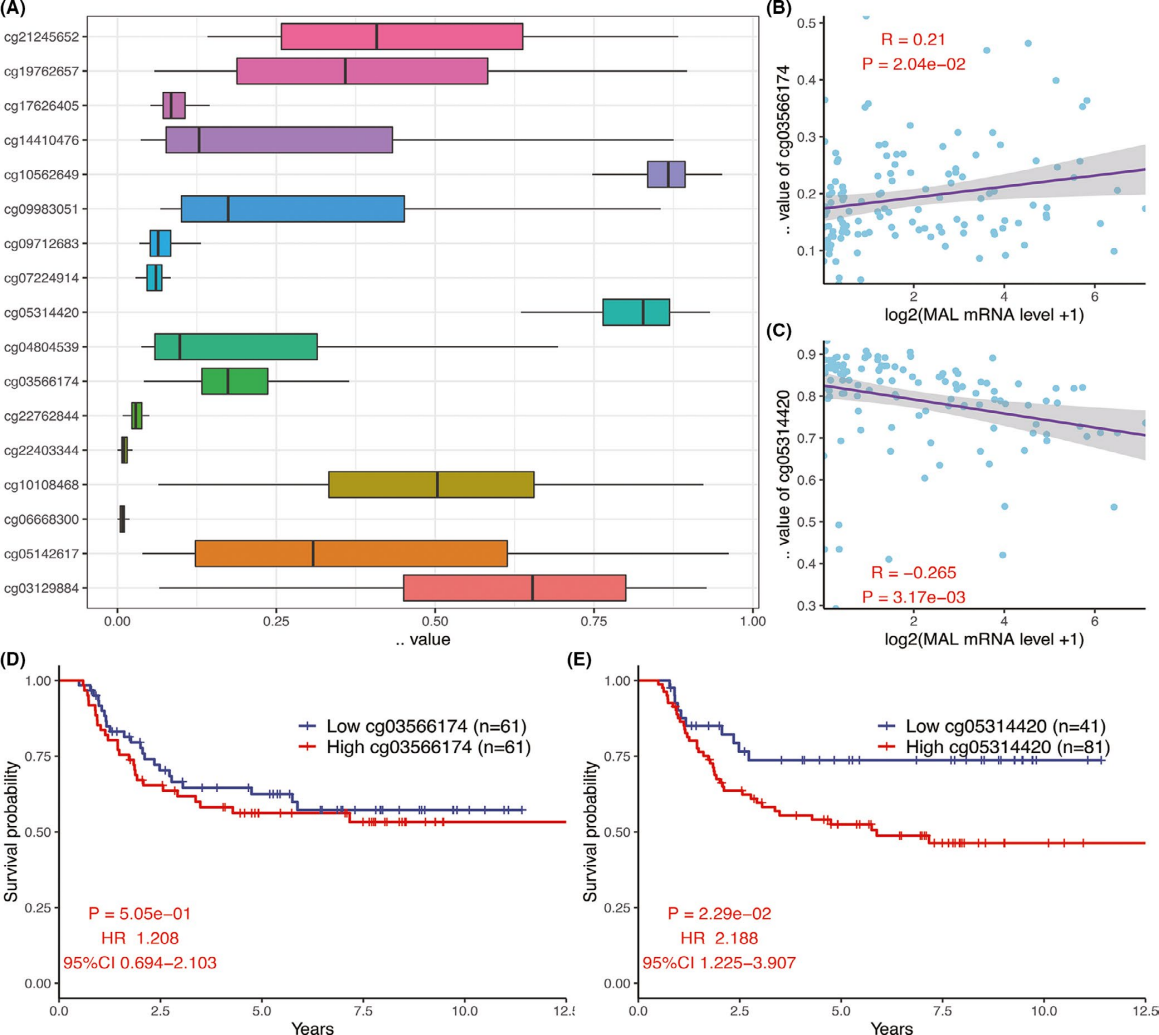
(A) The expression of *β* values at 17 methylation sites in the *MAL* gene. (B, C) Correlation analysis between expression of *MAL* and value of methylation. (D, E) KM survival analysis of cg03566174 and cg05314420 methylation sites in WT

We found that there were 17 methylation sites in the *MAL* gene. The *β* value of the methylation site in *the MAL* gene is shown in Figure 5A. The correlation analysis showed that *MAL* expression was related to cg03566174 (*R* = 0.21, *p* = 2.04E‐02, Figure 5B, Figure S1) and cg05314420 (*R* = −0.265, *p* = 3.17e‐03, Figure 5C, Figure S1) methylation sites. Kaplan–Meier analysis revealed that the cg05314420 methylation site of *MAL* was associated with OS (*p* = 2.29e‐02, HR = 2.188, 95% CI: 1.225–3.907, Figure 5D,E).

### 3.4 *MAL* expression and tumor microenvironment

**FIGURE 6:**
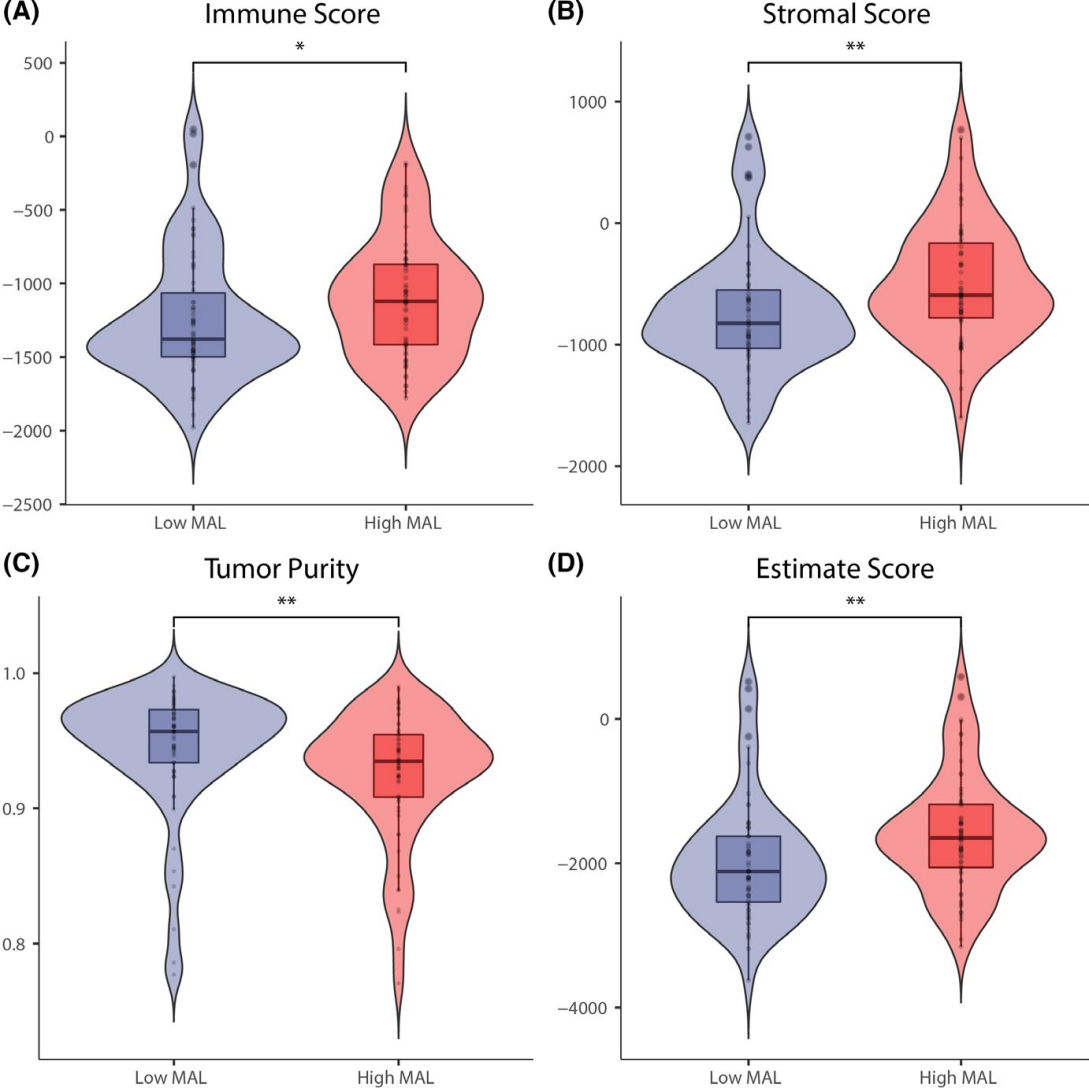
The relationship of between the expression file of *MAL* with immune score, stromal score, ESTIMATE score, and Tumor purity of tumor microenvironment. The high‐*MAL* group exhibited higher immune score, higher stromal score, higher ESTIMATE score, and lower tumor purity compared to low‐*MAL* group. *means *p* < 0.05, **means *p* < 518 0.01, and ***means *p* < 0.001. An ns means not statistically significant

**FIGURE 7:**
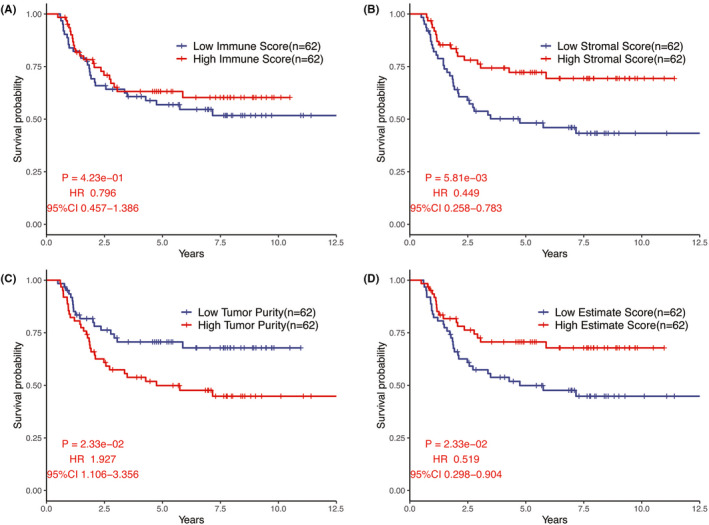
KM survival analysis of immune score, stromal score, ESTIMATE score, and tumor purity in patients with WT

The results of the immune microenvironment score indicated that the immune score, stromal score, and ESTIMATE score in the high‐*MAL* group were statistically higher than those in the low‐*MAL* group, while the tumor purity was opposite (*p* < 0.05, Figure 6A–D). KM analysis showed that the stromal score and ESTIMATE score presented protective factors, while tumor purity showed a risk factor, which indirectly reflected the low‐*MAL* group with a worse prognosis (Figure 7A–D).

**FIGURE 8:**
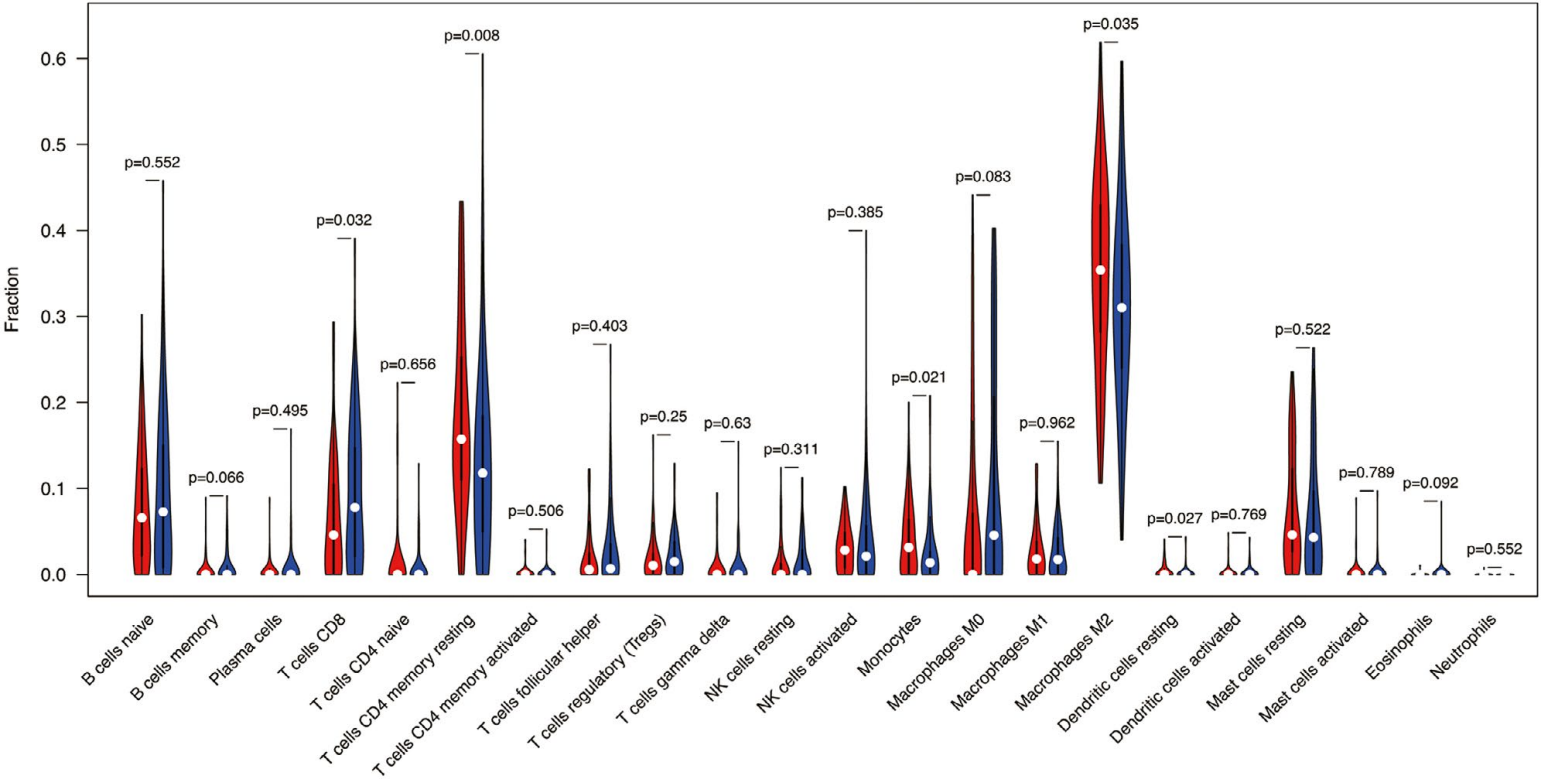
Differential infiltration analysis of 22 immune cell components in patients with WT. The red represents high‐*MAL* group, and blue represents low‐*MAL* group. The X axis represents the type of immune cells, and the Y axis represents the average level of immune cell infiltration in different *MAL* groups. *means *p* < 0.05, **means *p* < 0.01, and ***means *p* < 0.001. An ns means not statistically significant

Additionally, the infiltration analysis of 22 immune cell components of each WT sample revealed that CD8 T cells, resting CD4 memory T cells, monocytes, M2 macrophages, resting dendritic cells were significantly different between the high‐ and low‐*MAL* groups via Wilcoxon analysis(*p* < 0.05, Figure 8), which indicated that there were certain differences in immune cell infiltration in tumor microenvironment between high‐ and low‐*MAL* groups, which may affect the prognosis of WT patients to a certain extent.

### 3.5 Differential signaling pathways demonstrate through GSEA between the high‐ and low‐*MAL* groups

**FIGURE 9:**
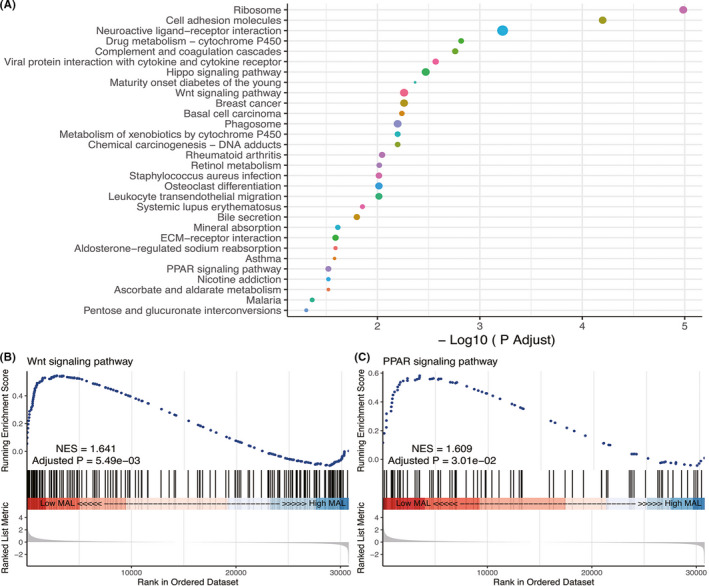
Parts of GSEA analysis results. Various signaling pathways associated with *MAL* are shown, including (B) Wnt signaling pathway and (C) PPAR signaling pathway

We performed GSEA between low‐ and high‐*MAL* groups and the result showed that some important signaling pathways existed differentially enriched between the two groups, such as Wnt signaling pathways and PPAR signaling pathways (NES > 1, *p* Adjust <0.05, Figure 9A–C).

## 4 DISCUSSION

As an integrated‐membrane protein with multiple biological functions and playing a significant role in cell differentiation and proliferation, *MAL* has been extensively studied in relation to numerous tumors.^20^, ^21^ *MAL* not only inhibited the progression of cancer,^15^, ^22^, ^23^ but in some cases, it also promoted the development of cancer.^17^, ^24^, ^25^, ^26^ Moreover, the role of *MAL* in the kidney has been mentioned in relevant studies.^21^, ^27^, ^28^ However, there have been few studies on the role of *MAL* in WT. In our study, we found that *MAL* was under‐expressed in WT, and it was not significantly correlated with age, gender, or stage. Meanwhile, RT‐qPCR in vitro also confirmed the low expression of *MAL* in WT, which provided us with a preliminary understanding of the role of *MAL* in WT.

Considering the difference of *MAL* expression between WT and adjacent normal tissues, the survival status difference of WT was further analyzed based on the grouping of *MAL* expression values. Meanwhile, the univariate and multivariate Cox analyses were also used to explore the influence of *MAL* expression on prognosis of WT and it was shown to be an independent prognostic factor in our analysis. Therefore, in the subsequent analysis, we conducted a series of explorations based on the expression difference of *MAL* in WT to understand the potential impact of *MAL* as a prognostic factor.

The level of gene methylation can affect the expression of genes, and methylation of *MAL* has been extensively demonstrated in a variety of cancers.^15^, ^16^, ^22^, ^23^, ^29^ Therefore, in order to explore the methylation level of *MAL* in WT, we used Pearson correlation analysis to investigate the relationship between *MAL* expression and methylation sites in WT. The results showed that the expression of *MAL* was closely related to the methylation sites of cg05314420 and cg03566174. It was worth noting that cg05314420 was positively correlated with the expression of *MAL*, while cg03566174 was negatively correlated with the expression of *MAL*. Therefore, the decrease of *MAL* expression may be the result of the combined effects of multiple methylation sites. Further, our analysis showed that methylation sites of cg05314420 could affect the OS of patients with WT, which indicated that the degree of methylation of *MAL* may be one of the reasons why *MAL* is a prognostic factor of WT. Unfortunately, the related methylation sites of *MAL* in WT had not been reported, but which would provide us with a new idea for the study of WT.

Not only the characteristics of the tumor itself, but also the microenvironment of the tumor affects the progression of the tumor. There is growing evidence showing that the tumor microenvironment is critical to the occurrence and prognosis of tumors.^30^, ^31^ A large number of studies have highlighted the presence of immunosuppressive microenvironments in WT^32^, ^33^, ^34^ and immune infiltration microenvironment may play an important role in the development of WT.^35^ Therefore, we further explored the difference of tumor microenvironment between high‐ and low‐*MAL* group by the ESTIMATE algorithm and CIBERSORT algorithm. The results of ESTIMATE algorithm showed that patients with low‐*MAL* expression turned out to have lower stromal score, immune, and ESTIMATE scores, while purity of tumor was the opposite. The patients with higher stromal score or lower tumor purity had more favorable prognostic. This is consistent with previous studies.^36^, ^37^ Meanwhile, the results of CIBERSORT algorithm also indicated that the expression of *MAL* was closely correlated with the infiltration of CD8 T cells, resting CD4 memory T cells, monocytes, M2 macrophages, and resting dendritic cells, which suggested to some extent that *MAL*, as a prognostic factor of WT, may be closely related to tumor microenvironment. Due to the low mutation load and immunogenicity of WT, it was not sensitive to most immunotherapy methods. However, because of the low toxicity of immunotherapy, the exploration of immunotherapy approaches for WT has never stopped.^35^, ^38^ Therefore, it was hoped that our study could bring some ideas for the follow‐up research of WT.

To investigate the differential activated signaling pathways between the high‐ and low‐*MAL* groups, we performed GSEA. The results showed that the differential activated signaling pathways between the high‐ and low‐*MAL* groups were important in cancer development, such as the Wnt signaling pathway and PPAP signaling pathway. The Wnt signaling pathway is a highly conserved pathway throughout evolution, and the complexity and function of the pathway is of vital importance in human embryonic development and growth.^39^, ^40^ Activation of the Wnt signaling pathway is very common in tumors and is abundant in other diseases.^41^, ^42^ In terms of cancer, Fan et al. demonstrated that RTL1 could promote melanoma proliferation by regulating the Wnt/β‐catenin signaling pathway^43^ and Korbut et al. also revealed the Wnt/β‐catenin signaling pathway regulated by Tiam1 could contribute the metastasis of thyroid cancer.^44^ In breast carcinoma, ALX4 has been shown to be a downregulated epigenetic tumor suppressor gene that could also inhibit the progression of breast cancer via the Wnt/β‐catenin pathway.^45^ Moreover, the mutations of related genes in the Wnt signaling pathway play a critical role in WT,^46^, ^47^, ^48^ which suggests the importance of the Wnt signaling pathway in WT. Peroxisome proliferator‐activated receptors (PPARs) are nuclear transcription factors that are related to cellular growth and differentiation, energy metabolism, insulin sensitization, and tumor regulation.^49^, ^50^ PPARs include three major members: PPARɑ, PPAR β/δ, and PPARγ, which have different tissue distribution and functions.^51^ In the kidney, PPARɑ regulates the homeostasis of energy metabolism^52^ and PPARγ maintains lipid and glucose homeostasis and is important for the control of renal function.^53^ Although little research has been done on the effect of the PPAR signaling pathway in the WT, our study would provide a novel view for the study of the WT and PPAP signaling pathways. The results of GSEA showed that multiple signaling pathways closely associated with tumorigenesis were differentially enriched between the high‐ and low‐*MAL* groups, which greatly demonstrated the different biological status between the high‐ and low‐ *MAL* groups, and further indicated the feasibility of *MAL* as a potential prognostic factor for WT.

This study comprehensively explored the potential of *MAL* as a prognosis factor for WT through complex bioinformatics analysis. Meanwhile, we also demonstrated that *MAL*, as a prognostic factor for WT, may be closely related to the tumor microenvironment. However, some limitations remain to be considered in our research. First, due to a lack of support from more in vitro and in vivo experiments, the credibility of tumor microenvironment‐related analysis is a challenge. Second, the data in our study comes from public databases, so there are unknown risks in grouping, blinding, and hidden bias. Therefore, although we have demonstrated the potential of *MAL* as a prognostic factor for WT in various ways, a prospective, well‐designed clinical trial is awaiting further validation of our findings.

## 5 CONCLUSION

In this study, we explored the potential of *MAL* as a prognosis factor for WT through related bioinformatics analysis. Meanwhile, we also demonstrated that *MAL*, as a prognostic factor for WT, may be closely related to the tumor microenvironment. Our results were expected to provide a new perspective for exploring prognostic factors of WT.

## CONFLICT OF INTEREST

All authors had read and approved to submit it to your journal. There were no conflicts of interest of any author in relation to the submission.

## AUTHOR CONTRIBUTIONS

All authors read and approved the final manuscript.

## ETHICS APPROVAL AND CONSENT TO PARTICIPATE

All procedures were approved by Institutional Review Boards of the First Affiliated Hospital of Guangxi Medical University. Written informed consents were obtained from patients involved in the study.

## Supporting information

Supplementary Material

Supplementary Material

## Data Availability

R 3.6.1 (http://www.r‐project.org/ ) was an open‐source software. The RNA‐FPKM data and clinical data of WT samples came from UCSC Xena (https://xenabrowser.net). GSE2712, GSE1115, and GSE73209 were downloaded from Gene Expression Omnibus (GEO. http://, www.ncbi.nlm.nih.gov/geo/). The methylation data of WT were downloaded from the Target database (https://ocg.cancer.gov/programs/target). The c5.bp.v6.2.entrez.gmt file came from Molecular Signatures Database (MSigDB, http://software.broadinstitute.org/gsea/ index. jsp).
