# Supplementary material for: Myelin and lymphocyte protein serves as a prognostic biomarker and is closely associated with the tumor microenvironment in the nephroblastoma
Source: Cancer Med. 2022 Jan 13;11(5):1427–38. doi: 10.1002/cam4.4542 (PMC8894696; doi:10.1002/cam4.4542)
Supplement: Supplementary file 1 — Supplementary Material [file CAM4-11-1427-s001.docx]

**0.pre-prepare**

if(!require("survival")) BiocManager::install("survival",update = F,ask = F)

if(!require("survminer")) BiocManager::install("survminer",update = F,ask = F)

if(!require("data.table")) BiocManager::install("data.table",update = F,ask = F)

if(!require("tidyverse")) BiocManager::install("tidyverse",update = F,ask = F)

if(!require("TCGAbiolinks")) BiocManager::install("TCGAbiolinks",update = F,ask = F)

if(!require("SummarizedExperiment")) BiocManager::install("SummarizedExperiment",update = F,ask = F)

if(!require("ggsignif")) BiocManager::install("ggsignif",update = F,ask = F)

if(!require("ggplot2")) BiocManager::install("ggplot2",update = F,ask = F)

if(!require("ggsci")) BiocManager::install("ggsci",update = F,ask = F)

if(!require("ggpubr")) BiocManager::install("ggpubr",update = F,ask = F)

if(!require("cowplot")) BiocManager::install("cowplot",update = F,ask = F)

if(!require("hgu133a.db")) BiocManager::install("hgu133a.db",update = F,ask = F)

if(!require("GEOquery")) BiocManager::install("GEOquery",update = F,ask = F)

if(!require("hgu133plus2.db")) BiocManager::install("hgu133plus2.db",update = F,ask = F)

if(!require("illuminaHumanv4.db")) BiocManager::install("illuminaHumanv4.db",update = F,ask = F)

if(!require("estimate")) BiocManager::install("estimate",update = F,ask = F)

if(!require("vioplot")) BiocManager::install("vioplot",update = F,ask = F)

if(!require("edgeR")) BiocManager::install("edgeR",update = F,ask = F)

if(!require("clusterProfiler")) BiocManager::install("clusterProfiler",update = F,ask = F)

if(!require("enrichplot")) BiocManager::install("enrichplot",update = F,ask = F)

if(!require("clusterProfiler")) BiocManager::install("clusterProfiler",update = F,ask = F)

**1.download data**

rm(list = ls())

##########download data

get_expression <- function(proj) {

query <- GDCquery(

project = proj,

data.category = "Transcriptome Profiling",

data.type = "Gene Expression Quantification",

workflow.type = "HTSeq - FPKM"

)

GDCdownload(query,directory = "TCGAbiolinksData/")

data <- GDCprepare(query,directory = "TCGAbiolinksData/")

load("Supp/GRCh37.Rda")

Symbol <- GRCh37 %>%

filter(type=="gene") %>%

dplyr::select(c(gene_name,gene_id))

exp <- assay(data) %>% as.data.frame() %>%

rownames_to_column(var="gene_id") %>%

inner_join(Symbol,by = "gene_id") %>%

dplyr::select(-"gene_id") %>%

dplyr::select('gene_name',everything()) %>%

filter('gene_name' != "NA") %>%

mutate(rowMean =rowMeans(.[grep("TCGA", names(.))])) %>%

arrange(desc(rowMean)) %>%

distinct(gene_name,.keep_all = T) %>%

dplyr::select(-rowMean)%>%

column_to_rownames(var = "gene_name")

return(exp)

}

RAWData <- get_expression("TARGET-WT")

##########download clinical infornation

if (!file.exists("TCGAbiolinksData/TARGET-WT.clinical.tsv.gz")) {

download.file("https://gdc-hub.s3.us-east-1.amazonaws.com/download/TARGET-WT.clinical.tsv.gz",

destfile = "TCGAbiolinksData/TARGET-WT.clinical.tsv.gz")

}

####Remove inappropriate samples

Samples <- as.character(c(colnames(RAWData)[substr(colnames(RAWData),18,19)=="11"],

colnames(RAWData)[substr(colnames(RAWData),18,19)=="01"]))

RAWData <- RAWData[,Samples]

Samples <- substr(Samples,1,20)

table(duplicated(Samples))

colnames(RAWData) <- Samples

WT_Clinic <- fread("TCGAbiolinksData/TARGET-WT.clinical.tsv.gz",header = T)

WT_Clinic <- WT_Clinic[!duplicated(WT_Clinic$sample_id),]

WT_Clinic <- column_to_rownames(WT_Clinic,var="sample_id")

WT_Clinic <- WT_Clinic[Samples[substr(Samples,18,19)=="01"],]

WT_Clinic <- WT_Clinic[!duplicated(rownames(WT_Clinic)),]

WT_Clinic <- WT_Clinic[,c(5,2,13,16,9,8)]

colnames(WT_Clinic) <- c("Age","Gender","Stage","Histologic","OS_Days","OS_Status")

save(RAWData,WT_Clinic,file = "RAWdataAnalysis.Rda")

########download GSE data

#######GSE11151

dir.create("GSE")

GSE11151 <- getGEO("GSE11151",AnnotGPL = F,getGPL = F,destdir = "GSE")

GSE11151_exp <- exprs(GSE11151$GSE11151_series_matrix.txt.gz)

probeset <- rownames(GSE11151_exp)

symbol <- annotate::lookUp(probeset,"hgu133plus2.db","SYMBOL")

symbol <- data.frame(probeset,"symbol"=as.vector(unlist(symbol)),stringsAsFactors = F)

GSE11151_exp <- as.data.frame(log2(GSE11151_exp+1)) %>%

rownames_to_column(var="probeset") %>%

inner_join(symbol,by = "probeset") %>%

dplyr::select(-"probeset") %>%

dplyr::select('symbol',everything()) %>%

filter(symbol!= 'NA') %>%

mutate(rowMean =rowMeans(.[grep("GSM", names(.))])) %>%

arrange(desc(rowMean)) %>%

distinct(symbol,.keep_all = T) %>%

dplyr::select(-rowMean) %>%

column_to_rownames(var = "symbol")

GSE11151_Clincal <- pData(GSE11151$GSE11151_series_matrix.txt.gz)

GSE11151_Clincal <- GSE11151_Clincal[,c(2,1)]

colnames(GSE11151_Clincal) <- c("GSM","Disease")

index <- c(grep("adult normal kidney",GSE11151_Clincal$Disease),

grep("fetal normal kidney",GSE11151_Clincal$Disease),

grep("Wilms' tumor",GSE11151_Clincal$Disease))

GSE11151_Clincal <- GSE11151_Clincal[index,]

GSE11151_exp <- GSE11151_exp[,as.character(GSE11151_Clincal$GSM)]

###########GSE2712

GSE2712 <- getGEO("GSE2712",AnnotGPL = F,getGPL = F,destdir = "GSE")

GSE2712_exp <- exprs(GSE2712$GSE2712_series_matrix.txt.gz)

probeset <- rownames(GSE2712_exp)

symbol <- annotate::lookUp(probeset,"hgu133a.db","SYMBOL")

symbol <- data.frame(probeset,"symbol"=as.vector(unlist(symbol)),stringsAsFactors = F)

GSE2712_exp <- as.data.frame(log2(GSE2712_exp+1)) %>%

rownames_to_column(var="probeset") %>%

inner_join(symbol,by = "probeset") %>%

dplyr::select(-"probeset") %>%

dplyr::select('symbol',everything()) %>%

filter(symbol!= 'NA') %>%

mutate(rowMean =rowMeans(.[grep("GSM", names(.))])) %>%

arrange(desc(rowMean)) %>%

distinct(symbol,.keep_all = T) %>%

dplyr::select(-rowMean) %>%

column_to_rownames(var = "symbol")

GSE2712_Clinical <- pData(GSE2712$GSE2712_series_matrix.txt.gz)

GSE2712_Clinical <- GSE2712_Clinical[,c(2,1)]

colnames(GSE2712_Clinical) <- c("GSM","Disease")

index <- c(grep("FK",GSE2712_Clinical$Disease),grep("WT",GSE2712_Clinical$Disease))

GSE2712_Clinical <- GSE2712_Clinical[index,]

GSE2712_exp <- GSE2712_exp[,as.character(GSE2712_Clinical$GSM)]

########GSE73209

GSE73209 <- getGEO("GSE73209",AnnotGPL = F,getGPL = F,destdir = "GSE")

GSE73209_exp <- exprs(GSE73209$GSE73209_series_matrix.txt.gz)

probeset <- rownames(GSE73209_exp)

symbol <- annotate::lookUp(probeset,"illuminaHumanv4.db","SYMBOL")

symbol <- data.frame(probeset,"symbol"=as.vector(unlist(symbol)),stringsAsFactors = F)

GSE73209_exp <- as.data.frame(log2(GSE73209_exp+1)) %>%

rownames_to_column(var="probeset") %>%

inner_join(symbol,by = "probeset") %>%

dplyr::select(-"probeset") %>%

dplyr::select('symbol',everything()) %>%

filter(symbol!= 'NA') %>%

mutate(rowMean =rowMeans(.[grep("GSM", names(.))])) %>%

arrange(desc(rowMean)) %>%

distinct(symbol,.keep_all = T) %>%

dplyr::select(-rowMean) %>%

column_to_rownames(var = "symbol")

GSE73209_Clinical <- pData(GSE73209$GSE73209_series_matrix.txt.gz)

GSE73209_Clinical <- GSE73209_Clinical[,c(2,32)]

colnames(GSE73209_Clinical) <- c("GSM","Disease")

index <- c(grep("adult kidney",GSE73209_Clinical$Disease),

grep("fetal kidney",GSE73209_Clinical$Disease),

grep("Wilms tumor",GSE73209_Clinical$Disease))

GSE73209_Clinical <- GSE73209_Clinical[index,]

GSE73209_exp <- GSE73209_exp[,as.character(GSE73209_Clinical$GSM)]

save(GSE11151_exp,GSE11151_Clincal,GSE2712_exp,GSE2712_Clinical,GSE73209_exp,GSE73209_Clinical,file = "GSERAWData.Rda")

**2.Figure1 Differentially expressed MAL**

rm(list = ls())

#####TARGET

load("RAWdataAnalysis.Rda")

MAL <- as.data.frame(t(log2(RAWData+1)["MAL",]))

MAL$Group <- c(rep("Paracancer",6),rep("Tumor",124))

p1 <- ggplot(data = MAL,aes(x=Group,y = MAL,fill=Group)) +

geom_boxplot(outlier.colour = "white",outlier.stroke = F) +

geom_point(size=0.8) +

scale_fill_aaas() +

geom_signif(comparisons = list(c("Paracancer","Tumor")),

textsize = 5,test = "t.test",map_signif_level = T) +

ylab(label = "Log2(MAL mRNA levels + 1)") +

xlab(label = NULL) +

ggtitle(label = "TARGET") +

theme(plot.title = element_text(hjust = 0.5),

panel.border = element_blank(),

axis.title.y = element_text(size=16),

axis.text = element_text(size=14),

axis.line = element_line(colour = "black",size = 0.6),

legend.position = "none")

##########

load("GSERAWData.Rda")

########GSE11151

GSE11151MAL <- as.data.frame(t(GSE11151_exp["MAL",GSE11151_Clincal$GSM]))

GSE11151MAL$Group <- c(rep("ANK",3),rep("FNK",2),rep("WT",4))

p2 <- ggplot(data = GSE11151MAL,aes(x=Group,y = MAL,fill=Group)) +

geom_boxplot(outlier.colour = "white",outlier.stroke = F) +

geom_point(size=0.8) +

scale_fill_aaas() +

geom_signif(comparisons = list(c("FNK","WT"),c("ANK","WT")),

step_increase = 0.06,test = "t.test",map_signif_level = T,

textsize = 5) +

ylab(label = "Log2(MAL mRNA levels + 1)") +

xlab(label = NULL) +

ggtitle(label = "GSE11151") +

theme(plot.title = element_text(hjust = 0.5),

panel.border = element_blank(),

axis.title.y = element_text(size=16),

axis.text = element_text(size=14),

axis.line = element_line(colour = "black",size = 0.6),

legend.position = "none")

##########GSE2712

GSE2712MAL <- as.data.frame(t(GSE2712_exp["MAL",GSE2712_Clinical$GSM]))

GSE2712MAL$Group <- c(rep("FNK",3),rep("WT",18))

p3 <- ggplot(data = GSE2712MAL,aes(x=Group,y = MAL,fill=Group)) +

geom_boxplot(outlier.colour = "white",outlier.stroke = F) +

geom_point(size=0.8) +

scale_fill_aaas() +

geom_signif(comparisons = list(c("FNK","WT")),

test = "t.test",map_signif_level = T,

textsize = 5) +

ylab(label = "Log2(MAL mRNA levels + 1)") +

xlab(label = NULL) +

ggtitle(label = "GSE2712") +

theme(plot.title = element_text(hjust = 0.5),

panel.border = element_blank(),

axis.title.y = element_text(size=16),

axis.text = element_text(size=14),

axis.line = element_line(colour = "black",size = 0.6),

legend.position = "none")

##########GSE73209

GSE73209MAL <- as.data.frame(t(GSE73209_exp["MAL",GSE73209_Clinical$GSM]))

GSE73209MAL$Group <- c(rep("ANK",2),rep("FNK",4),rep("WT",32))

p4 <- ggplot(data = GSE73209MAL,aes(x=Group,y = MAL,fill=Group)) +

geom_boxplot(outlier.colour = "white",outlier.stroke = F) +

geom_point(size=0.8) +

scale_fill_aaas() +

geom_signif(comparisons = list(c("FNK","WT"),c("ANK","WT")),

step_increase = 0.06, test = "t.test",map_signif_level = T,

textsize = 5) +

ylab(label = "Log2(MAL mRNA levels + 1)") +

xlab(label = NULL) +

ggtitle(label = "GSE73209") +

theme(plot.title = element_text(hjust = 0.5),

panel.border = element_blank(),

axis.title.y = element_text(size=16),

axis.text = element_text(size=14),

axis.line = element_line(colour = "black",size = 0.6),

legend.position = "none")

Figure2 <- plot_grid(p1,p3,p2,p4,align = "hv",

labels = c("A","B","C","D"),label_size = 18)

save_plot(Figure2,filename = "Figure2.pdf",base_width = 9,base_height = 10)

**3.Figure3 Relationship of MAL and clinical information**

rm(list = ls())

load("RAWdataAnalysis.Rda")

WT_Clinic <- as.data.frame(WT_Clinic) %>%

mutate(Gender = case_when(Gender == "Female" ~ "Female",

Gender == "Male" ~ "Male"),

Stage = case_when(Stage == "I" ~ "I",

Stage == "II" ~ "II",

Stage %in% c("III", "IIIB","IIIB/V") ~ "III",

Stage %in% c("IV","IV/V") ~ "IV")) %>%

mutate(Gender = factor(Gender, levels = c("Male", "Female")),

Stage = factor(Stage, levels = c( "I", "II", "III", "IV")))

WT_Clinic$Age <- ifelse(WT_Clinic$Age>6*365,">6","<=6")

WT_Clinic$Stage <- ifelse(WT_Clinic$Stage %in% c("I","II"),"I/II","III/IV")

WT_Clinic <- inner_join(rownames_to_column(WT_Clinic,var="Samples"),

rownames_to_column(as.data.frame(t(log2(RAWData+1))),var = "Samples")[,c("Samples","MAL")])

#######Age

p1 <- ggplot(WT_Clinic,aes(x=Age,y=MAL,color=Age)) +

geom_boxplot(aes(fill=Age),alpha=0.2,na.rm = F,width=0.3,

position = position_dodge(width = 1)) +

geom_violin(aes(fill=Age),alpha=0.2,na.rm = F,trim = F,

position = position_dodge(width = 1))+

geom_point(aes(fill=Age),size=1,na.rm = T,

position = position_jitterdodge(dodge.width = 1,jitter.width = 0.05,

jitter.height = 0.5)) +

stat_boxplot(geom = "errorbar",width=0.05,position =

position_dodge(width = 1)) +

geom_signif(comparisons = list(c("<=6",">6")),test = "wilcox.test",

y_position = 9,textsize = 6) +

scale_color_aaas() +

scale_fill_aaas() +

ylab(label = "Log2(MAL mRNA Expression+1)") +

theme(panel.border = element_blank(),

axis.title = element_text(size=16),

axis.text = element_text(size=14),

axis.line = element_line(colour = "black",size = 0.6),

legend.position = "none")

#######Gender

p2 <- ggplot(WT_Clinic,aes(x=Gender,y=MAL,color=Gender)) +

geom_boxplot(aes(fill=Gender),alpha=0.2,na.rm = F,width=0.3,

position = position_dodge(width = 1)) +

geom_violin(aes(fill=Gender),alpha=0.2,na.rm = F,trim = F,

position = position_dodge(width = 1))+

geom_point(aes(fill=Gender),size=1,na.rm = T,

position = position_jitterdodge(dodge.width = 1,jitter.width = 0.2,

jitter.height = 0.5)) +

stat_boxplot(geom = "errorbar",width=0.05,position =

position_dodge(width = 1)) +

geom_signif(comparisons = list(c("Male","Female")),test = "wilcox.test",

y_position = 9.2,textsize = 6) +

scale_color_aaas() +

scale_fill_aaas() +

ylab(label = "Log2(MAL mRNA Expression+1))") +

theme(panel.border = element_blank(),

axis.title = element_text(size=16),

axis.text = element_text(size=14),

axis.line = element_line(colour = "black",size = 0.6),

legend.position = "none")

#####Stage

p3 <- ggplot(WT_Clinic,aes(x=Stage,y=MAL,color=Stage)) +

geom_boxplot(aes(fill=Stage),alpha=0.2,na.rm = F,width=0.3,

position = position_dodge(width = 1)) +

geom_point(aes(fill=Stage),size=1.5,na.rm = T,

position = position_jitterdodge(jitter.width = 0.04)) +

geom_violin(aes(fill=Stage),alpha=0.2,na.rm = F,trim = F,

position = position_dodge(width = 1))+

stat_boxplot(geom = "errorbar",width=0.05,position =

position_dodge(width = 1)) +

geom_signif(comparisons = list(c("I/II","III/IV")),test = "wilcox.test",

y_position = 9.2,textsize = 6) +

scale_color_aaas() +

scale_fill_aaas() +

ylab(label = "Log2(MAL mRNA Expression+1)") +

theme(panel.border = element_blank(),

axis.title = element_text(size=16),

axis.text = element_text(size=14),

axis.line = element_line(colour = "black",size = 0.6),

legend.position = "none")

###Histologic

p4 <- ggplot(WT_Clinic,aes(x=Histologic,y=MAL,color=Histologic)) +

geom_boxplot(aes(fill=Histologic),alpha=0.2,na.rm = F,width=0.3,

position = position_dodge(width = 1)) +

geom_point(aes(fill=Histologic),size=1.5,na.rm = T,

position = position_jitterdodge(jitter.width = 0.04)) +

geom_violin(aes(fill=Histologic),alpha=0.2,na.rm = F,trim = F,

position = position_dodge(width = 1))+

stat_boxplot(geom = "errorbar",width=0.05,position =

position_dodge(width = 1)) +

geom_signif(comparisons = list(c("DAWT","FHWT")),test = "wilcox.test",

y_position = 9.2,textsize = 6) +

scale_color_aaas() +

scale_fill_aaas() +

ylab(label = "Log2(MAL mRNA Expression+1)") +

theme(panel.border = element_blank(),

axis.title = element_text(size=16),

axis.text = element_text(size=14),

axis.line = element_line(colour = "black",size = 0.6),

legend.position = "none")

Figure3 <- plot_grid(p1,p2,p3,nrow = 1,align = "hv",labels = c("A","B","C"))

save_plot(Figure3,filename = "Figure3.pdf",base_width = 12,base_height = 5)

**4.Figure4 KM Analysis and Table2 Cox analysis**

rm(list = ls())

load("RAWdataAnalysis.Rda")

WT_Clinic <- as.data.frame(WT_Clinic) %>%

mutate(Gender = case_when(Gender == "Female" ~ "Female",

Gender == "Male" ~ "Male"),

Stage = case_when(Stage == "I" ~ "I",

Stage == "II" ~ "II",

Stage %in% c("III", "IIIB","IIIB/V") ~ "III",

Stage %in% c("IV","IV/V") ~ "IV")) %>%

mutate(Gender = factor(Gender, levels = c("Male", "Female")),

Stage = factor(Stage, levels = c( "I", "II", "III", "IV")))

WT_Clinic$Age <- ifelse(WT_Clinic$Age>6*365,">6","<=6")

WT_Clinic$Stage <- ifelse(WT_Clinic$Stage %in% c("I","II"),"I/II","III/IV")

WT_Clinic <- inner_join(rownames_to_column(WT_Clinic,var="Samples"),

rownames_to_column(as.data.frame(t(log2(RAWData+1))),var = "Samples")[,c("Samples","MAL")])

WT_Clinic$subtype <- ifelse(WT_Clinic$MAL > median(WT_Clinic$MAL),"HighMAL","LowMAL")

WT_Clinic$subtype <- factor(WT_Clinic$subtype,levels = c("LowMAL","HighMAL"))

WT_Clinic[,c(2,4,5)] <- lapply(WT_Clinic[,c(2,4,5)], as.factor)

######KM Analysis

kmfit <- surv_fit(Surv(WT_Clinic$OS_Days/365,WT_Clinic$OS_Status=="Dead")~subtype, data = WT_Clinic)

sdf <- survdiff(Surv(WT_Clinic$OS_Days/365,WT_Clinic$OS_Status=="Dead")~subtype, data = WT_Clinic)

table(WT_Clinic$subtype)

Figure4 <- ggsurvplot(kmfit,legend.labs=c("Low MAL(n=62)","High MAL (n=62)"),

pval = F,cumcensor = T)$p +

scale_color_aaas() +

theme_classic(base_size = 14) +

theme(legend.position = c(0.7,0.8),

legend.background = element_blank(),

legend.text = element_text(size = 15),

legend.title = element_blank()) +

xlab(label = "Years") +

annotate("text",x=3,y=0.18,size=5,color="red",

label=paste0("P = ",format(surv_pvalue(kmfit)$pval,scientific = T,digits = 3),

"\nHR ",round((sdf$obs[2]/sdf$exp[2])/(sdf$obs[1]/sdf$exp[1]),3),

"\n95%CI ",paste0(round(exp(log(round((sdf$obs[2]/sdf$exp[2])/(sdf$obs[1]/sdf$exp[1]),3)) - qnorm(0.975)*sqrt(1/sdf$exp[2]+1/sdf$exp[1])),3),"-",

round(exp(log(round((sdf$obs[2]/sdf$exp[2])/(sdf$obs[1]/sdf$exp[1]),3)) + qnorm(0.975)*sqrt(1/sdf$exp[2]+1/sdf$exp[1])),3))))

save_plot(Figure4,filename = "Figure4.pdf",base_width = 6,base_height =6 )

############Cox analysis

CreatTable <- function(CoxAnalysis,Values,data){

CoxRes <- summary(CoxAnalysis)

CoxRes1 <- data.frame(Characteristics = unlist(lapply(Values,function(i){

c(i,levels(data[,i]))

})))

CoxRes2 <- data.frame(Characteristics = unlist(lapply(Values,function(i){c(levels(data[,i])[2])})),

"HR(95%CI)" = paste0(round(CoxRes$coefficients[,"exp(coef)"],3)," (",

round(CoxRes$conf.int[,3],2),'-',

round(CoxRes$conf.int[,4],2),")"),

P = format(CoxRes$coefficients[,'Pr(>|z|)'],scientific = T,digits = 3))

CoxRes <- left_join(CoxRes1,CoxRes2)

CoxRes[CoxRes$Characteristics %in% unlist(lapply(Values,function(i){c(levels(data[,i])[1])})),"HR.95.CI."] <- "Reference"

return(CoxRes)

}

mySur <- Surv(WT_Clinic$OS_Days/365,WT_Clinic$OS_Status=="Dead")

UnivariateCoxAnalysis <- function(Val){

formal <- as.formula(paste0("mySur~",Val))

cph <- coxph(formal,data = WT_Clinic)

UnKC <- CreatTable(cph,Val,WT_Clinic)

return(UnKC)

}

UnivarCoxres <- lapply(c("Age","Gender","Stage","Histologic","subtype"), function(i) UnivariateCoxAnalysis(i))

UnivarCoxres <- plyr::ldply(UnivarCoxres,data.frame)

MultivarCoxph <- coxph(as.formula(paste0("mySur~",paste0(c("Gender","Stage",'subtype'),collapse = "+"))), data =WT_Clinic)

MultivarCoxres <- CreatTable(MultivarCoxph,c("Gender","Stage",'subtype'),WT_Clinic)

Table2 <- left_join(UnivarCoxres,MultivarCoxres,by='Characteristics')

write.csv(Table2,file = "Table2.csv",row.names = F)

**5.Figure5 MAL and methylation**

rm(list = ls())

load("Supp/MALMethBeta.Rda")

load("RAWdataAnalysis.Rda")

MALMethBeta <- MALMethBeta[,22:ncol(MALMethBeta)]

MALMethBeta["Mean",] <- colMeans(MALMethBeta)

WT_Clinic <- inner_join(rownames_to_column(WT_Clinic,var="Samples"),

rownames_to_column(as.data.frame(t(log2(RAWData+1))),var = "Samples")[,c("Samples","MAL")])

WT_Clinic$subtype <- ifelse(WT_Clinic$MAL > median(WT_Clinic$MAL),"HighMAL","LowMAL")

WT_Clinic$subtype <- factor(WT_Clinic$subtype,levels = c("LowMAL","HighMAL"))

MALMethBeta <- inner_join(WT_Clinic,rownames_to_column(as.data.frame(t(MALMethBeta)),var = "Samples"))

MALMethBetaboxplot <- reshape2::melt(MALMethBeta[,10:26])

p1 <- ggplot(MALMethBetaboxplot,aes(x=variable,y=value,fill=variable)) +

geom_boxplot(outlier.alpha = 0) +

coord_flip() +

xlab(label = NULL) +

ylab(label = 'β value') +

theme_bw(base_size = 14) +

theme(legend.position = "none")

##########Correlation analysis

corres <- lapply(colnames(MALMethBeta)[10:26], function(fl){

PearsonRe <- cor.test(MALMethBeta[,fl],MALMethBeta[,"MAL"],

method = "pearson",exact = F)

R <- round(PearsonRe$estimate[[1]],3)

Pval <- format(PearsonRe$p.value,scientific = T,digits = 3)

result <- data.frame(R=R,P=Pval)

rownames(result) <- fl

return(result)

})

S1 <- do.call(rbind,corres)

S1 <- rownames_to_column(S1,var="methylation sites")

write.csv(S1,file = "S1.csv",row.names = F,quote = F)

#########cg03566174

p2 <- ggplot(MALMethBeta,aes(x=MAL,y=cg03566174)) +

geom_point(size=2,color="skyblue") +

scale_x_continuous(expand = c(0,0))+

scale_y_continuous(expand = c(0,0))+

stat_smooth(method='lm',color="purple")+

ylab(label = "β value of cg03566174")+

xlab(label="log2(MAL mRNA level +1)") +

theme_classic(base_size = 14) +

annotate("text",label= paste0('R = ',corres["cg03566174","R"],

"\nP = ",corres["cg03566174","P"]),

x=2,y=0.45,vjust=0.5,size=5,color="red")

MALMethBeta$cg03566174 <- ifelse(MALMethBeta$cg03566174 > median(MALMethBeta$cg03566174),"Highcg03566174",

"Lowcg03566174")

MALMethBeta$cg03566174 <- factor(MALMethBeta$cg03566174,levels = c("Lowcg03566174","Highcg03566174"))

kmfit <- surv_fit(Surv(MALMethBeta$OS_Days/365,MALMethBeta$OS_Status=="Dead")~cg03566174, data = MALMethBeta)

sdf <- survdiff(Surv(MALMethBeta$OS_Days/365,MALMethBeta$OS_Status=="Dead")~cg03566174, data = MALMethBeta)

p3 <- ggsurvplot(kmfit,legend.labs=c("Low cg03566174 (n=61)","High cg03566174 (n=61)"),

pval = F,cumcensor = T)$p +

scale_color_aaas() +

scale_x_continuous(expand = c(0,0)) +

theme_classic(base_size = 14) +

theme(legend.position = c(0.7,0.8),

legend.background = element_blank(),

legend.text = element_text(size = 15),

legend.title = element_blank()) +

xlab(label = "Years") +

annotate("text",x=2,y=0.15,size=5,color="red",

label=paste0("P = ",format(surv_pvalue(kmfit)$pval,scientific = T,digits = 3),

"\nHR ",round((sdf$obs[2]/sdf$exp[2])/(sdf$obs[1]/sdf$exp[1]),3),

"\n95%CI ",paste0(round(exp(log(round((sdf$obs[2]/sdf$exp[2])/(sdf$obs[1]/sdf$exp[1]),3)) - qnorm(0.975)*sqrt(1/sdf$exp[2]+1/sdf$exp[1])),3),"-",

round(exp(log(round((sdf$obs[2]/sdf$exp[2])/(sdf$obs[1]/sdf$exp[1]),3)) + qnorm(0.975)*sqrt(1/sdf$exp[2]+1/sdf$exp[1])),3))))

#########cg05314420

p4 <- ggplot(MALMethBeta,aes(x=MAL,y=cg05314420)) +

geom_point(size=2,color="skyblue") +

scale_x_continuous(expand = c(0,0))+

scale_y_continuous(expand = c(0,0))+

stat_smooth(method='lm',color="purple")+

ylab(label = "β value of cg05314420")+

xlab(label="log2(MAL mRNA level +1)") +

theme_classic(base_size = 14) +

annotate("text",label= paste0('R = ',corres["cg05314420","R"],

"\nP = ",corres["cg05314420","P"]),

x=2,y=0.35,vjust=0.5,size=5,color="red")

MALMethBeta$cg05314420 <- ifelse(MALMethBeta$cg05314420 > mean(MALMethBeta$cg05314420),"Highcg05314420",

"Lowcg05314420")

MALMethBeta$cg05314420 <- factor(MALMethBeta$cg05314420,levels = c("Lowcg05314420","Highcg05314420"))

kmfit <- surv_fit(Surv(MALMethBeta$OS_Days/365,MALMethBeta$OS_Status=="Dead")~cg05314420, data = MALMethBeta)

sdf <- survdiff(Surv(MALMethBeta$OS_Days/365,MALMethBeta$OS_Status=="Dead")~cg05314420, data = MALMethBeta)

p5 <- ggsurvplot(kmfit,legend.labs=c("Low cg05314420 (n=41)","High cg05314420 (n=81)"),

pval = F,cumcensor = T)$p +

scale_color_aaas() +

scale_x_continuous(expand = c(0,0)) +

theme_classic(base_size = 14) +

theme(legend.position = c(0.7,0.8),

legend.background = element_blank(),

legend.text = element_text(size = 15),

legend.title = element_blank()) +

xlab(label = "Years") +

annotate("text",x=2,y=0.15,size=5,color="red",

label=paste0("P = ",format(surv_pvalue(kmfit)$pval,scientific = T,digits = 3),

"\nHR ",round((sdf$obs[2]/sdf$exp[2])/(sdf$obs[1]/sdf$exp[1]),3),

"\n95%CI ",paste0(round(exp(log(round((sdf$obs[2]/sdf$exp[2])/(sdf$obs[1]/sdf$exp[1]),3)) - qnorm(0.975)*sqrt(1/sdf$exp[2]+1/sdf$exp[1])),3),"-",

round(exp(log(round((sdf$obs[2]/sdf$exp[2])/(sdf$obs[1]/sdf$exp[1]),3)) + qnorm(0.975)*sqrt(1/sdf$exp[2]+1/sdf$exp[1])),3))))

p6 <- plot_grid(p2,p4,nrow = 2,labels = c("B","C"),label_size = 18)

p7 <- plot_grid(p1,p6,nrow = 1,rel_widths = c(2:1),labels = c("A",NULL,NULL),label_size = 18)

p8 <- plot_grid(p3,p5,nrow = 1,labels = c("D","E"),label_size = 18)

Figure5 <- plot_grid(p7,p8,nrow = 2,rel_heights = c(1,0.6),align = "hv")

save_plot(Figure5,filename = "Figure5.pdf",base_width = 13,base_height = 12)

**6.Figure6-7 Relationship of MAL and immuncescore**

rm(list = ls())

############EstimateAnslysis

load("RAWdataAnalysis.Rda")

WT_Clinic <- inner_join(rownames_to_column(WT_Clinic,var="Samples"),

rownames_to_column(as.data.frame(t(log2(RAWData+1))),var = "Samples")[,c("Samples","MAL")])

WT_Clinic$subtype <- ifelse(WT_Clinic$MAL > median(WT_Clinic$MAL),"High MAL","Low MAL")

WT_Clinic$subtype <- factor(WT_Clinic$subtype,levels = c("Low MAL","High MAL"))

write.table(as.data.frame(RAWData[,WT_Clinic$Samples]), "RAWDataExpr.txt", quote = FALSE, sep = "\t")

filterCommonGenes(input.f = "RAWDataExpr.txt", output.f = "RAWDataExpr.gct", id = "GeneSymbol")

estimateScore("RAWDataExpr.gct", "RAWDataExprscore.gct", platform = 'affymetrix')

estimatescore <- read.table("RAWDataExprscore.gct",sep = "\t", skip = 2,header = T)

rownames(estimatescore) <- estimatescore[, 1]

estimatescore <- as.data.frame(t(estimatescore[, -c(1,2)]))

rownames(estimatescore) <- gsub("\\.",'-',rownames(estimatescore))

estimatescore <- inner_join(WT_Clinic,rownames_to_column(estimatescore,var = "Samples"))

write.csv(estimatescore,file = "Originational data of Figure7.csv",quote = F,row.names = F)

#########ImmunceScore

p1 <- ggplot(data = estimatescore,aes(x=subtype,y=ImmuneScore,fill=subtype)) +

geom_boxplot(aes(fill=subtype),alpha=0.4,width=0.3) +

geom_violin(aes(fill=subtype),alpha=0.4,trim = F,width=1,position = position_dodge(width = 0.9)) +

geom_point(size=0.7,alpha=0.2) +

geom_signif(comparisons = list(c("Low MAL","High MAL")),y_position = 400,

test = "wilcox.test",map_signif_level = T,

textsize = 5) +

xlab(label = NULL) +

ylab(label = NULL) +

ggtitle(label="Immune Score") +

scale_fill_manual(values = c(ggsci::pal_aaas()(2)[1],ggsci::pal_aaas()(2)[2])) +

theme_classic(base_size = 14) +

theme(legend.position = "none",

plot.title = element_text(hjust = 0.5),

axis.line = element_line(colour = "black",size=0.3))

######StromalScore

p2 <- ggplot(data = estimatescore,aes(x=subtype,y=StromalScore,fill=subtype)) +

geom_boxplot(aes(fill=subtype),alpha=0.4,width=0.3) +

geom_violin(aes(fill=subtype),alpha=0.4,trim = F,width=1,position = position_dodge(width = 0.9)) +

geom_point(size=0.7,alpha=0.2) +

scale_fill_manual(values = c(ggsci::pal_aaas()(2)[1],ggsci::pal_aaas()(2)[2])) +

geom_signif(comparisons = list(c("Low MAL","High MAL")),y_position = 1200,

test = "wilcox.test",map_signif_level = T,

textsize = 5) +

xlab(label = NULL) +

ylab(label = NULL) +

ggtitle(label="Stromal Score") +

theme_classic(base_size = 14) +

theme(legend.position = "none",

plot.title = element_text(hjust = 0.5),

axis.line = element_line(colour = "black",size=0.3))

######TumorPurity

p3 <- ggplot(data = estimatescore,aes(x=subtype,y=TumorPurity,fill=subtype)) +

geom_boxplot(aes(fill=subtype),alpha=0.4,width=0.22,outlier.colour = "white") +

geom_violin(aes(fill=subtype),alpha=0.4,trim = F,width=1,position = position_dodge(width = 0.9)) +

geom_point(size=0.5,alpha=0.2) +

geom_signif(comparisons = list(c("Low MAL","High MAL")),y_position = 1.03,

test = "wilcox.test",map_signif_level = T,

textsize = 5) +

xlab(label = NULL) +

ylab(label = NULL) +

scale_fill_manual(values = c(ggsci::pal_aaas()(2)[1],ggsci::pal_aaas()(2)[2])) +

ggtitle(label="Tumor Purity") +

theme_classic(base_size = 14) +

theme(legend.position = "none",

plot.title = element_text(hjust = 0.5),

axis.line = element_line(colour = "black",size=0.3))

#####ESTIMATEScore

p4 <- ggplot(data = estimatescore,aes(x=subtype,y=ESTIMATEScore,fill=subtype)) +

geom_boxplot(aes(fill=subtype),alpha=0.4,width=0.3) +

geom_violin(aes(fill=subtype),alpha=0.4,trim = F,width=0.8) +

geom_point(size=0.7,alpha=0.2) +

geom_signif(comparisons = list(c("Low MAL","High MAL")),y_position = 1200,

test = "wilcox.test",map_signif_level = T,

textsize = 5) +

xlab(label = NULL) +

ylab(label = NULL) +

scale_fill_manual(values = c(ggsci::pal_aaas()(2)[1],ggsci::pal_aaas()(2)[2])) +

ggtitle(label="Estimate Score") +

theme_classic(base_size = 14) +

theme(legend.position = "none",

plot.title = element_text(hjust = 0.5),

axis.line = element_line(colour = "black",size=0.3))

Figure6 <- plot_grid(p1,p2,p3,p4,labels = c('A',"B","C","D"),label_size = 18)

save_plot(Figure6,filename = "Figure6.pdf",base_width = 9,base_height = 9)

#########ImmunceScore KM analysis

estimatescore$ImmuneScore <- ifelse(estimatescore$ImmuneScore > median(estimatescore$ImmuneScore),

"High Immune Score","Low Immune Score")

estimatescore$ImmuneScore <- factor(estimatescore$ImmuneScore,levels = c("Low Immune Score","High Immune Score"))

kmfit <- surv_fit(Surv(estimatescore$OS_Days/365,estimatescore$OS_Status=="Dead")~ImmuneScore, data = estimatescore)

sdf <- survdiff(Surv(estimatescore$OS_Days/365,estimatescore$OS_Status=="Dead")~ImmuneScore, data = estimatescore)

p5 <- ggsurvplot(kmfit,legend.labs=c("Low Immune Score(n=62)","High Immune Score(n=62)"),

pval = F,cumcensor = T)$p +

scale_color_aaas() +

scale_x_continuous(expand = c(0,0)) +

theme_classic(base_size = 14) +

theme(legend.position = c(0.7,0.8),

legend.background = element_blank(),

legend.text = element_text(size = 15),

legend.title = element_blank()) +

xlab(label = "Years") +

annotate("text",x=2,y=0.15,size=5,color="red",

label=paste0("P = ",format(surv_pvalue(kmfit)$pval,scientific = T,digits = 3),

"\nHR ",round((sdf$obs[2]/sdf$exp[2])/(sdf$obs[1]/sdf$exp[1]),3),

"\n95%CI ",paste0(round(exp(log(round((sdf$obs[2]/sdf$exp[2])/(sdf$obs[1]/sdf$exp[1]),3)) - qnorm(0.975)*sqrt(1/sdf$exp[2]+1/sdf$exp[1])),3),"-",

round(exp(log(round((sdf$obs[2]/sdf$exp[2])/(sdf$obs[1]/sdf$exp[1]),3)) + qnorm(0.975)*sqrt(1/sdf$exp[2]+1/sdf$exp[1])),3))))

#########StromalScore KM analysis

estimatescore$StromalScore <- ifelse(estimatescore$StromalScore > median(estimatescore$StromalScore),

"High Stromal Score","Low Stromal Score")

estimatescore$StromalScore <- factor(estimatescore$StromalScore,levels = c("Low Stromal Score","High Stromal Score"))

kmfit <- surv_fit(Surv(estimatescore$OS_Days/365,estimatescore$OS_Status=="Dead")~StromalScore, data = estimatescore)

sdf <- survdiff(Surv(estimatescore$OS_Days/365,estimatescore$OS_Status=="Dead")~StromalScore, data = estimatescore)

p6 <- ggsurvplot(kmfit,legend.labs=c("Low Stromal Score(n=62)","High Stromal Score(n=62)"),

pval = F,cumcensor = T)$p +

scale_color_aaas() +

scale_x_continuous(expand = c(0,0)) +

theme_classic(base_size = 14) +

theme(legend.position = c(0.7,0.8),

legend.background = element_blank(),

legend.text = element_text(size = 15),

legend.title = element_blank()) +

xlab(label = "Years") +

annotate("text",x=2,y=0.15,size=5,color="red",

label=paste0("P = ",format(surv_pvalue(kmfit)$pval,scientific = T,digits = 3),

"\nHR ",round((sdf$obs[2]/sdf$exp[2])/(sdf$obs[1]/sdf$exp[1]),3),

"\n95%CI ",paste0(round(exp(log(round((sdf$obs[2]/sdf$exp[2])/(sdf$obs[1]/sdf$exp[1]),3)) - qnorm(0.975)*sqrt(1/sdf$exp[2]+1/sdf$exp[1])),3),"-",

round(exp(log(round((sdf$obs[2]/sdf$exp[2])/(sdf$obs[1]/sdf$exp[1]),3)) + qnorm(0.975)*sqrt(1/sdf$exp[2]+1/sdf$exp[1])),3))))

#########TumorPurity KM analysis

table(estimatescore$TumorPurity > median(estimatescore$TumorPurity))

estimatescore$TumorPurity <- ifelse(estimatescore$TumorPurity > median(estimatescore$TumorPurity),

"High Tumor Purity","Low Tumor Purity")

estimatescore$TumorPurity <- factor(estimatescore$TumorPurity,levels = c("Low Tumor Purity","High Tumor Purity"))

kmfit <- surv_fit(Surv(estimatescore$OS_Days/365,estimatescore$OS_Status=="Dead")~TumorPurity, data = estimatescore)

sdf <- survdiff(Surv(estimatescore$OS_Days/365,estimatescore$OS_Status=="Dead")~TumorPurity, data = estimatescore)

p7 <- ggsurvplot(kmfit,legend.labs=c("Low Tumor Purity(n=62)","High Tumor Purity(n=62)"),

pval = F,cumcensor = T)$p +

scale_color_aaas() +

scale_x_continuous(expand = c(0,0)) +

theme_classic(base_size = 14) +

theme(legend.position = c(0.7,0.8),

legend.background = element_blank(),

legend.text = element_text(size = 15),

legend.title = element_blank()) +

xlab(label = "Years") +

annotate("text",x=2,y=0.15,size=5,color="red",

label=paste0("P = ",format(surv_pvalue(kmfit)$pval,scientific = T,digits = 3),

"\nHR ",round((sdf$obs[2]/sdf$exp[2])/(sdf$obs[1]/sdf$exp[1]),3),

"\n95%CI ",paste0(round(exp(log(round((sdf$obs[2]/sdf$exp[2])/(sdf$obs[1]/sdf$exp[1]),3)) - qnorm(0.975)*sqrt(1/sdf$exp[2]+1/sdf$exp[1])),3),"-",

round(exp(log(round((sdf$obs[2]/sdf$exp[2])/(sdf$obs[1]/sdf$exp[1]),3)) + qnorm(0.975)*sqrt(1/sdf$exp[2]+1/sdf$exp[1])),3))))

#########EstimateScore KM analysis

estimatescore$ESTIMATEScore <- ifelse(estimatescore$ESTIMATEScore> median(estimatescore$ESTIMATEScore),

"High Estimate Score","Low Estimate Score")

estimatescore$ESTIMATEScore <- factor(estimatescore$ESTIMATEScore,levels = c("Low Estimate Score","High Estimate Score"))

kmfit <- surv_fit(Surv(estimatescore$OS_Days/365,estimatescore$OS_Status=="Dead")~ESTIMATEScore, data = estimatescore)

sdf <- survdiff(Surv(estimatescore$OS_Days/365,estimatescore$OS_Status=="Dead")~ESTIMATEScore, data = estimatescore)

p8 <- ggsurvplot(kmfit,legend.labs=c("Low Estimate Score(n=62)","High Estimate Score(n=62)"),

pval = F,cumcensor = T)$p +

scale_color_aaas() +

scale_x_continuous(expand = c(0,0)) +

theme_classic(base_size = 14) +

theme(legend.position = c(0.7,0.8),

legend.background = element_blank(),

legend.text = element_text(size = 15),

legend.title = element_blank()) +

xlab(label = "Years") +

annotate("text",x=2,y=0.15,size=5,color="red",

label=paste0("P = ",format(surv_pvalue(kmfit)$pval,scientific = T,digits = 3),

"\nHR ",round((sdf$obs[2]/sdf$exp[2])/(sdf$obs[1]/sdf$exp[1]),3),

"\n95%CI ",paste0(round(exp(log(round((sdf$obs[2]/sdf$exp[2])/(sdf$obs[1]/sdf$exp[1]),3)) - qnorm(0.975)*sqrt(1/sdf$exp[2]+1/sdf$exp[1])),3),"-",

round(exp(log(round((sdf$obs[2]/sdf$exp[2])/(sdf$obs[1]/sdf$exp[1]),3)) + qnorm(0.975)*sqrt(1/sdf$exp[2]+1/sdf$exp[1])),3))))

Figure7 <- plot_grid(p5,p6,p7,p8,labels = c('A',"B","C","D"),label_size = 18)

save_plot(Figure7,filename = "Figure7.pdf",base_width = 12.5,base_height = 10)

**7.Figure8 Relationship of 22 immune cell and MAL**

rm(list = ls())

############

load("RAWdataAnalysis.Rda")

WT_Clinic <- inner_join(rownames_to_column(WT_Clinic,var="Samples"),

rownames_to_column(as.data.frame(t(log2(RAWData+1))),var = "Samples")[,c("Samples","MAL")])

WT_Clinic$subtype <- ifelse(WT_Clinic$MAL > median(WT_Clinic$MAL),"High MAL","Low MAL")

WT_Clinic$subtype <- factor(WT_Clinic$subtype,levels = c("Low MAL","High MAL"))

write.table(rbind(ID=colnames(RAWData[,WT_Clinic$Samples]),log2(RAWData[,WT_Clinic$Samples]+1)),file="RAWCIBERSORT.txt",sep="\t",quote=F,col.names=F)

########22 immune cell calculation

###waite for a minutes

source("Supp/CIBERSORT.R")

if (!file.exists("CIBERSOFTScore.Rda")){

set.seed(112)

CIBERSORTScore<- CIBERSORT("Supp/LM22.txt", "RAWCIBERSORT.txt",perm=1000, QN=TRUE)

save(CIBERSORTScore,file = 'CIBERSORTScore.Rda')

unlink("./CIBERSORT-Results.txt")

}else{

message("load from disk")

load(file = 'CIBERSORTScore.Rda')

}

CIBERSORTScore <- CIBERSORTScore[,1:22]

CIBERSORTvioplotdata <- rbind(CIBERSORTScore[rownames(CIBERSORTScore) %in% WT_Clinic$Samples[WT_Clinic$subtype=="High MAL"],],

CIBERSORTScore[rownames(CIBERSORTScore) %in% WT_Clinic$Samples[WT_Clinic$subtype=="Low MAL"],])

pdf("Figure8.pdf",height=8,width=15)

par(las=1,mar=c(10,6,3,3))

x=c(1:ncol(CIBERSORTvioplotdata))

y=c(1:ncol(CIBERSORTvioplotdata))

plot(x,y,

xlim=c(0,63),ylim=c(min(CIBERSORTvioplotdata),max(CIBERSORTvioplotdata)+0.02),

main="",xlab="", ylab="Fraction",

pch=21,

col="white",

xaxt="n")

High <- 62

Low <- 62

for(i in 1:ncol(CIBERSORTvioplotdata)){

HighData=CIBERSORTvioplotdata[1:High,i]

LowData=CIBERSORTvioplotdata[(High+1):(High+Low),i]

vioplot(HighData,at=3*(i-1),lty=1,add = T,col = 'red')

vioplot(LowData,at=3*(i-1)+1,lty=1,add = T,col = 'blue')

wilcoxTest=wilcox.test(HighData,LowData)

p=round(wilcoxTest$p.value,3)

mx=max(c(LowData,HighData))

lines(c(x=3*(i-1)+0.2,x=3*(i-1)+0.8),c(mx,mx))

text(x=3*(i-1)+0.5,y=mx+0.02,labels=ifelse(p<0.001,paste0("p<0.001"),paste0("p=",p)),cex = 0.8)

text(seq(1,64,3),-0.05,xpd = NA,labels=colnames(CIBERSORTvioplotdata),cex = 0.9,srt = 45,pos=2)

}

dev.off()

**8.Figure9 GSEA analysis**

rm(list = ls())

####

load("RAWdataAnalysis.Rda")

WT_Clinic <- inner_join(rownames_to_column(WT_Clinic,var="Samples"),

rownames_to_column(as.data.frame(t(log2(RAWData+1))),var = "Samples")[,c("Samples","MAL")])

WT_Clinic$subtype <- ifelse(WT_Clinic$MAL > median(WT_Clinic$MAL),"High MAL","Low MAL")

WT_Clinic$subtype <- factor(WT_Clinic$subtype,levels = c("Low MAL","High MAL"))

RAWData <- RAWData[,WT_Clinic$Samples]

group_list <- factor(WT_Clinic$subtype,levels = c("Low MAL","High MAL"))

#########identification of a order gene list

DGElist <- DGEList(counts = RAWData,group = factor(group_list))

DGElist <- calcNormFactors(DGElist,method = "TMM")

DGElist <- estimateCommonDisp(DGElist, verbose=TRUE)

DGElist <- estimateTagwiseDisp(DGElist)

et <- exactTest(DGElist)

MALnrDEG <- topTags(et,n = nrow(DGElist))$table

#####gseKEGG analysis

mRNALog <- data.frame("SYMBOL"=rownames(MALnrDEG),"LogFC"= as.numeric(MALnrDEG$logFC))

anno <- bitr(mRNALog$SYMBOL,fromType = "SYMBOL",toType = "ENTREZID",OrgDb = "org.Hs.eg.db")

mRNALog <- merge(mRNALog,anno,by="SYMBOL")

mRNALog_sort <- mRNALog[order(mRNALog$LogFC,decreasing = T),]

geneList <- mRNALog_sort$LogFC

names(geneList) <- mRNALog_sort$ENTREZID

C5 <- read.gmt("Supp/c5.bp.v6.2.entrez.gmt")

set.seed(112)

egmt <- gseKEGG(geneList)

KEGGRes <- as.data.frame(egmt)

KEGGRes <- KEGGRes[abs(KEGGRes$NES)>1.6&KEGGRes$p.adjust < 0.05,]

p1 <- ggplot(KEGGRes,aes(y=-log10(p.adjust),x=reorder(Description,-p.adjust))) +

geom_point(aes(color=Description,size=setSize)) +

coord_flip() +

theme_bw(base_size = 18) +

xlab(label = NULL) +

ylab(label =" - Log10 ( P Adjust)") +

theme(legend.position = "none",

panel.border = element_blank(),

axis.line = element_line(colour = "black",size = 0.5))

p2 <- gseaplot2(egmt,c("hsa04310"),title = "Wnt signaling pathway",base_size = 12,

ES_geom = "dot",color = pal_lancet(palette = "lanonc")(6)[1]) +

geom_text(aes(label="Low MAL <<<<< ------------------------------------ >>>>> High MAL",

x=0.55,y=0.37))+

geom_text(label= paste0("NES = ",round(KEGGRes["hsa04310",5],3)),

aes(x=0.25,y=0.6),size=5) +

geom_text(label= paste0("Adjusted P = ", format(KEGGRes["hsa04310",7],scientific = T,digits = 3)),

aes(x=0.25,y=0.55),size=5)

p3 <- gseaplot2(egmt,c("hsa03320"),title = "PPAR signaling pathway",base_size = 12,

ES_geom = "dot",color = pal_lancet(palette = "lanonc")(6)[1]) +

geom_text(aes(label="Low MAL <<<<< ------------------------------------ >>>>> High MAL",x=0.55,y=0.37))+

geom_text(label= paste0("NES = ",round(KEGGRes["hsa03320",5],3)),

aes(x=0.25,y=0.6),size=5) +

geom_text(label= paste0("Adjusted P = ", format(KEGGRes["hsa03320",7],scientific = T,digits = 3)),

aes(x=0.25,y=0.55),size=5)

p4 <- plot_grid(p2,p3,align = "hv",labels = c("B","C"),label_size = 18)

Figure9 <- plot_grid(p1,p4,ncol = 1,labels = "A",rel_heights = c(1.2,0.8),label_size = 18)

save_plot(Figure9,filename = "Figure9.pdf",base_width = 13,base_height = 11.5)
